# Analysis of Mutagenesis in the *Escherichia coli tdk* Gene: The Nucleoid Protein HNS Is Required for a Dramatic Cisplatin‐Induced Hotspot

**DOI:** 10.1002/em.70054

**Published:** 2026-05-11

**Authors:** Dana Sorensen, Tara Mata, Ananya Sridharan, Mallika Mathew, Emily Sagastume, Natasha Sutkin, Katherine Douglas, Mila Daniel, Angela Hung, Alina Garmash, Annette Hsieh, Jamie Elizabeth Ortega, Jeffrey H. Miller

**Affiliations:** ^1^ Department of Microbiology, Immunology, and Molecular Genetics, and the Molecular Biology Institute University of California, and the David Geffen School of Medicine Los Angeles California USA

**Keywords:** bacterial systems, mutational hotspots, *tdk*

## Abstract

We demonstrate the utility of the *tdk* reporter gene system, by showing its ability to not only analyze mutational hotspots, but also to allow the analysis of both weak and strong mutator or mutagen effects. It can detect large and small insertions and deletions, as well as base substitutions. We previously defined cisplatin (CPT)‐induced hotspots in *tdk*. One extraordinary hotspot for G:C‐ > T:A transversions at base pair (bp) 499 is in a region of the gene that appears to be mutationally prone, suggesting that the conformation of the DNA in that region may play a major role in elevating mutation rates. Here, we examined CPT‐induced mutations in a derivative of the starting wild‐type strain that lacks the nucleoid binding protein HNS, which is involved in the folding and compaction of the *E. coli* chromosome. In this strain background the extraordinary CPT‐induced hotspot disappears, pinpointing the importance of DNA conformation in mutation rates at certain positions. We further show that this A:T‐rich gene is a magnet for transposable elements.

## 1 Introduction

Seymour Benzer first defined “hotspots” in 1961, as sites that are more mutationally prone than the average site (Benzer 1961). In some cases the hotspots are dramatically more mutable. Although nearest neighbors can play an important role in mutations rates (e.g., Coulondre and Miller 1977; Miller 1985; Horsfall et al. 1990; Rodriguez and Loechler 1991; Nilsen et al. 1995; Hatahet et al. 1998; Foster et al. 2018), in many examples there are vast differences in the mutation rates at sites with the identical nearest neighbor bases (e.g., Miller 1985; see Discussion in Fernandez et al. 2020). Why some of these sites are “hot” and others not still remains an enigma. Therefore, it is critical to examine the larger sequence context of individual sites. In this respect, the more different sequence contexts that can be compared, the more likely we are to be able to identify the principles that lead to different mutation rates at different sites. We have recently characterized the *tdk*‐thymidine deoxykinase reporter system that includes 375 mutations at 264 of the 618 bp long gene (Young et al. 2024; Douglas et al. 2025). We have shown that hotspots from a variety of mutators and mutagens tend to occur in distinct regions of the *tdk* gene, as we have also shown for base substitutions in the *thyA*‐thymidylate synthase reporter system (Mashiach et al. 2021). We detected an extraordinary hotspot among the cisplatin (CPT) induced mutations in *tdk* (Young et al. 2024). Here we show that this hotspot depends on at least one nucleoid binding protein, HNS.

The *tdk* gene is AT rich (58% AT, 42% GC) in contradistinction to *E. coli* in general (48% AT, 52% GC), and to other widely used reporter genes (*lacI* 44% AT, *thyA* 49% AT). This raises the possibility of finding different results with certain mutagens and mutators. Here, we have extended the work with *tdk* to look at indels and transposable element integrations, as well as characterizing additional base substitution mutagens and mutators. Due to its AT richness, the *tdk* gene serves as a magnet for transposable element insertions, as reported in other systems (e.g., Tu and Cohen 1980). We also find that nucleoid proteins such as factor for inversion stimulation (FIS), histone‐like protein (HU‐alpha), integration host factor (IHF), and heat‐stable nucleoid structuring protein (HNS), have a significant effect on transpositions into the chromosomal *tdk* gene, as others have found for transpositions of IS1 into extrachromosomal elements (Shiga et al. 2001). This underscores the important role that DNA conformation can play in these events.

## 2 Materials and Methods

### 2.1 *E. coli* Strains

The wild‐type strain is BW25113 (Datsenko and Wanner 2000), which we have used previously (e.g., Ang et al. 2016; Young et al. 2024; see below). It is the starting strain for the KEIO collection, described in Baba et al. (2006). This strain is *lacI* ^*q*^ *rrnB*_*T14*_ *ΔlacZ*_*WJ16*_ *hsdR514 ΔaraBAD*_*AH33*_ *ΔrhaBAD*_*LD78*_. The MutD, FIS, HNS, IHF, HU‐alpha, and AlkB‐deficient strains used here are from the Keio collection. CC107 (Cupples et al. 1990) is: *ara ∆ (gpt‐lac)5 thi/F′128 lacIZ proA* ^*+*^ *B* ^+^. CC107 also carries a frameshift mutation in *lacZ* on the F′ plasmid. We constructed an *argE*::Tn10 derivative of CC107 by P1 transduction from CAG12185 (Singer et al. 1989). DE372 (Ennis et al. 1985) was a gift from Roger Woodgate. It is *lexA51*(Def) *sulA211 thi‐1 ∆ (gpt‐lac)5 ilv (Ts) mtl‐1 rpsL31 umuC122::Tn5*. MG1655 (Wanner and Boline 1990) is *phoM*(wt) *arcA1655 fnr‐1655*. The MG1655 *sodA, sodB* double deletion mutant derivative was a gift from Karin Chonoles Imlay and James Imlay.

### 2.2 Media

The following media (Miller 1972, 1992) were used: LB (10 g tryptone, 5 g yeast extract, 10 g NaCl per liter), and Minimal A buffer (10.5 g K_2_HPO_4_, 4.5 g KH_2_PO_4_, 1 g (NH_4_)_2_SO_4_, 0.5 g sodium citrate⋅2H_2_O per liter).

### 2.3 Growth Conditions and Mutagenesis Treatment

Unless otherwise stated, all genetic methods are as described by Miller (1972, 1992).

CPT (cisplatin) treatment: Overnight cultures grown in a 37° incubator were used to seed “overday” cultures by inoculating 250 μL into each of five separate 5 mL LB cultures that were then incubated in a 37° water bath for 3–4 h. After placing on ice for 5 min, the cultures were pooled and spun down, the liquid discarded and the pellets washed in minimal A buffer (unsupplemented minimal A), recentrifuged and resuspended in half the initial LB volume in minimal A buffer. A 2 mg/mL CPT stock was prepared in water and shaken vigorously for 1.5 h. The CPT stock was added to resuspended cultures to achieve a final concentration of 100 μg/mL, and incubated for 60 min at 37°. In all cases (NQO, EMS, CPT), the cells were spun down and resuspended into 2.5 mL minimal A buffer. These cultures were titered for survival. Outgrowth cultures were seeded with 0.2 mL of this resuspension in 5 mL LB and grown overnight at 37° on a rotor at 50 rpm (0.44 g) before plating. The treatments with CPT yielded survivals of 45%–70%.

MMS treatment: Cells were treated with MMS exactly as done for CPT, except that 0.03 mL MMS was added to 2 mL of washed cultures in place of CPT.

ICR treatment: Experiments with ICR191 were carried out in minimal A medium supplemented with 10 mL of 20% glucose, 1 mL of 1 M MgSO_4_, and 0.5 mL 0f 1% thiamine hydrochloride and 1% LB per liter. ICR191 was added to a concentration of 10 μg/mL.

For mutator strains: A culture grown overnight in an incubator at 37°C without additional aeration was used to seed sets of cultures. We diluted the cultures (10^−4^ dilution) and then added 20 μL (approximately 500–800 cells) to 2 mL of LB. These were then grown overnight at 37°C on a rotor at 50 rpm.

### 2.4 Determination of Mutant Frequencies

The cells grown as indicated above were plated on LB plates with or without 100 ng/mL azidothymidine (AZT; Young et al. 2024). The plates were scored after 24 h. The frequencies of AZT‐resistant mutants were determined as described previously (Young et al. 2024). Briefly, mutant frequency (*f*) was determined as the median frequency from a set of cultures (*N* = number of cultures). 95% confidence limits were determined according to Dixon and Massey Jr (1969). The frequencies are given in Table 1.

**TABLE 1:** *tdk* mutant frequencies (*f*) of *E. coli* using wild‐type and HNS‐deficient strains.

Strain	Condition	Time treated	Number of cultures	*tdk* median mutant frequency *f* (10^−8^)^a^
BW25113	—	—	32	101 (53–133)
HNS	—	—	30	62 (46–78)
BW25113	100 μg/mL CPT	30 min	8	1596 (1089–2661)
HNS	100 μg/mL CPT	30 min	10	2588 (1587–2993)

*Note: tdk* mutant frequencies (*f*).

^a^ Values in parentheses are 95% confidence limits.

### 2.5 Chromosomal DNA Isolation and Sequencing

Chromosomal DNA was isolated from overnight cultures of each mutant after single colony purification by streaking. We only picked one AZT‐resistant mutant per culture. The PCR tests were carried out as colony PCR reactions. The *tdk* gene was PCR‐amplified from genomic DNA using Taq polymerase (Invitrogen) and sets of primers which allowed us to sequence directly from the PCR product. The primer sequences were: tdk F (forward) 22mer 5′—CAAGGCTTCGTAAGGGAGAACG—3′, and tdk R (reverse) 21mer.

5′—CTGCCGAGAAGGGTATATAGC—3′. The sequencing primer was tdk F. The tdk F primer extends from 120 bases to 98 bases upstream of the 5′ end of the gene. The reverse primer extends from 72 bases to 93 bases downstream of the 3′ end of the gene.

PCR thermal cycling conditions were as follows: initial denaturation at 94 deg. C for 2 min; 30 cycles of 94 deg. C for 30 s, 58 deg. C for 30 s, and 72 deg. C for 1 min; followed by a final extension at 72 deg. C for 7 min and a 4 deg. C hold.

The unpurified PCR product was outsourced to Laragen (Culver City) for purification with exoSap (Affymetrix) and Sanger sequencing.

### 2.6 Chemicals

AZT, methyl methanesulfonate (MMS), and cisplatin (CPT) were purchased from Sigma (St. Louis, MO). ICR191 was purchased from Polysciences Inc. (Warrington, PA).

## 3 Results

### 3.1 Effect of DNA Conformation on Mutational Hotspots

**FIGURE 1:**
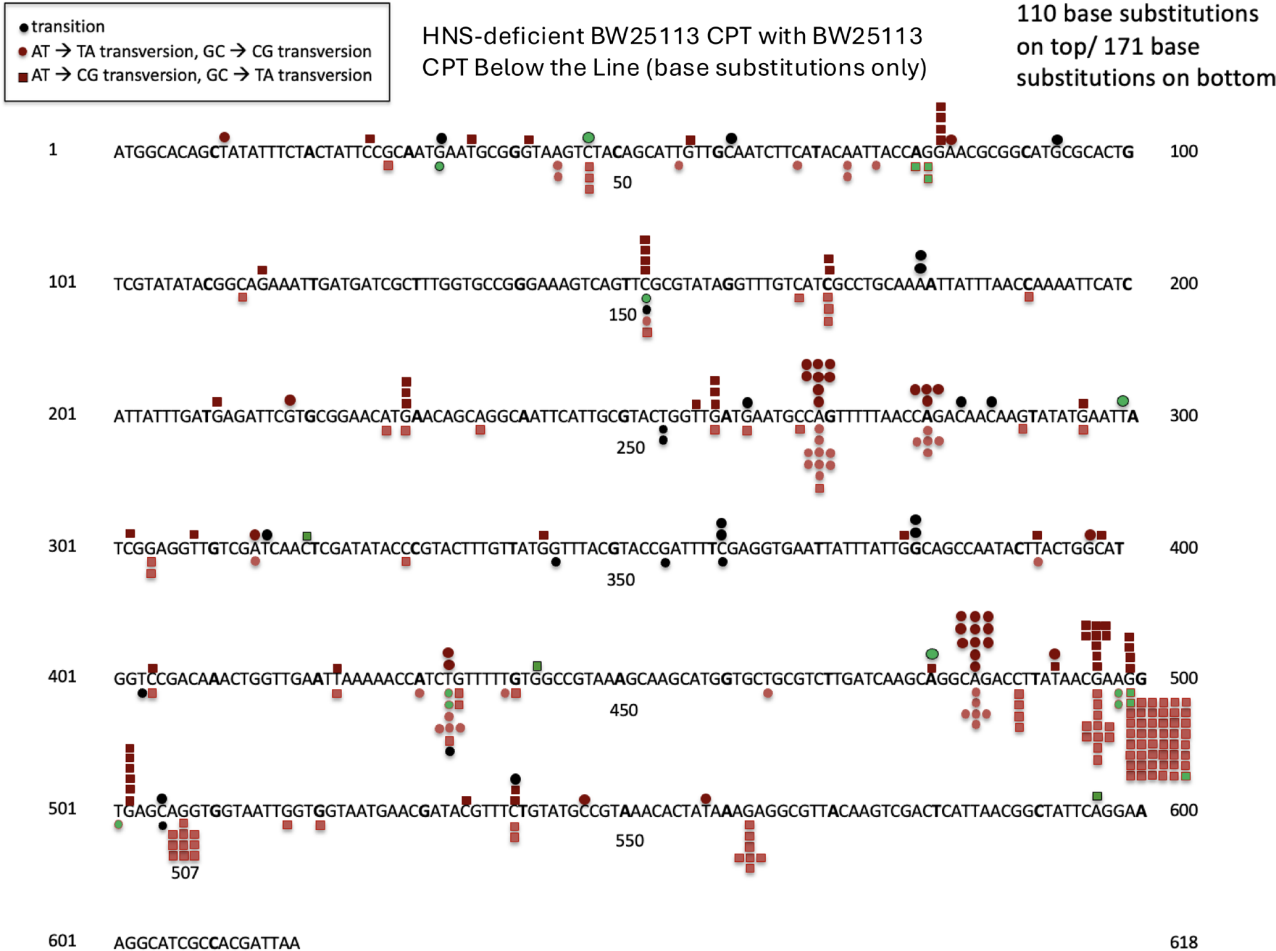
Distribution of CPT‐induced base substitution mutations in the *tdk* gene in BW25113 and its HNS‐deficient derivative. The BW25113 distribution is shown below the sequence “line,” and the HNS derivative distribution is shown above the line. Tandem double mutations are indicated by neighboring green squares. HNS‐deficient BW25113 CPT with BW25113 CPT below the line.

We used a derivative of strain BW25113 from the Keio collection (Baba et al. 2006), in which the *hns* gene has been deleted. CPT induces AZT‐resistance significantly over the unmutagenized background in both the starting strain and its *hns* derivative (Table 1; see also Young et al. 2024). We sequenced the *tdk* gene in 140 independent AZT‐resistant mutants. Base substitutions represented 110 of these mutations, the remainder being small insertions or deletions. The spectrum of the 110 base substitutions is displayed in Figure 1 and is compared with the spectrum we previously reported for CPT‐induced mutations in the starting BW25113 wild‐type strain (Young et al. 2024). The starting strain shows several well represented sites with G:C‐ > T:A or A:T‐ > T:A mutations, but the outstanding feature is an extraordinary G:C‐ > T:A hotspot at bp 499 at which 48 of 171 base substitutions (28%) occur. However, in the HNS‐deficient derivative of BW2511 the 499 site is represented by 3.6% of the base substitution mutations, with four occurrences out of 110 base substitution mutations, despite an expectation of 31 occurrences if 28% of the 110 mutations were at the 499 site. Based on this difference in percentage, mutations at the hotspot site would be reduced by 7.7‐fold. Moreover, the ratio of occurrences at the 499 site to the nearby G:C‐ > T:A site at 496 is 48:11 in the wild‐type BW25113 strain, but 4:9 in the *hns* strain (a 9.8‐fold difference). Likewise, the ratio of mutations at 499 to the A:T‐ > T:A mutations at 484 is 48:9 in the wild‐type, but 4:15 in the *hns* strain (a 20‐fold difference). These results indicate the very frequent G:C‐ > T:A transversions induced by CPT at 499 are dependent on the presence of a functional HNS protein and that the strong hotspot is a product of the HNS‐mediated DNA conformation at that point.

### 3.2 Transposon Element Magnet

**FIGURE 2:**
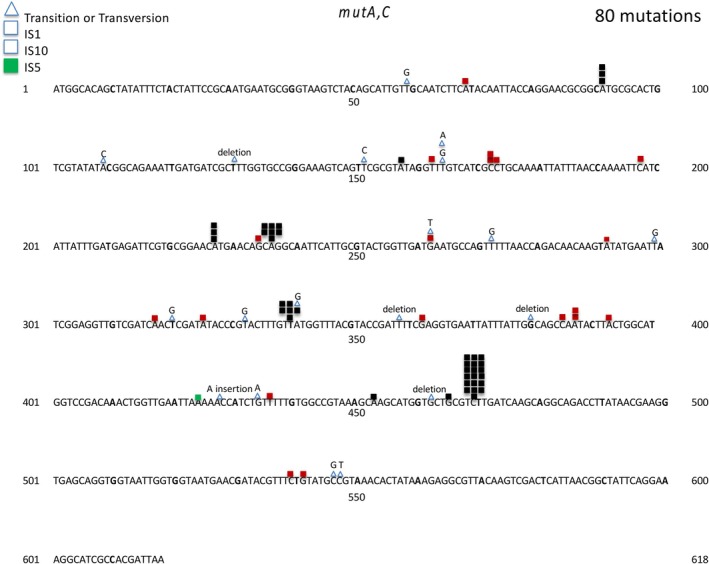
Distribution of spontaneous mutations in the *tdk* gene in CC107mutA, mutC. Insertion sequences (IS1, IS5, Tn10) are indicated by a square, and base substitutions by a triangle.

**FIGURE 3:**
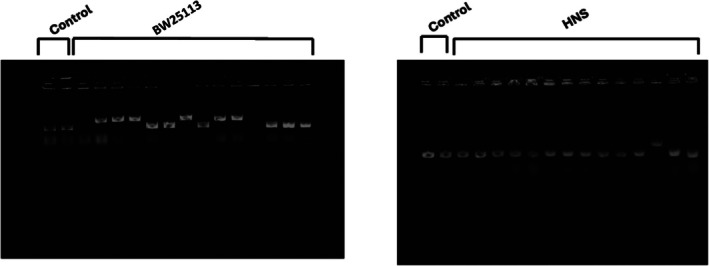
Example of a gel of PCR products in *tdk* from AZT resistant mutants. Here 6 of 12 mutants in the wild‐type BW25113 have insertions the size of IS1, and only 1 of 14 from an *hns* derivative of BW25113 has an IS1 size insertion.

We have previously reported the spectrum of spontaneous mutations in the *tdk* gene for two strains (CC107; BW25113; Young et al. 2024). Table 2 summarizes the results, and reveals that 24% of the 83 mutations that result in *tdk* inactivation are insertion elements. These are principally IS1insertions, with some IS3 elements. We determined the identity of all insertions by sequencing from the front end of the *tdk* gene and far enough into the inserted element to allow identification. We then utilized identified insertions as length standards in subsequent gel measurements. During the examination of a strain derived from CC107, with a weak base substitution mutator (*mutA,C*; Michaels et al. 1990), we were surprised to find the spectrum of spontaneous mutations shown in Figure 2. Namely, 50% of the mutations were Tn10 transpositions into *tdk*. Altogether, 76% of the 80 mutations were due to transposable elements. A Tn10 element near the *mutC* locus moved frequently into the AT rich *tdk* gene, even without selection for the tetracycline resistance encoded by the Tn10. The attraction of AT rich regions to transposable elements has been known for some time (e.g., Tu and Cohen 1980). The 618 bp *tdk* gene is 58% AT. Moreover, the *tdk* gene is within a 1400 bp region that is 59% AT, ranging from 700 bp prior to *tdk* to 100 bp after *tdk*. The Tn10 insertions clearly cluster at favored sites (Figure 2), and the site preferences of Tn10 have been well documented (Bender and Kleckner 1992). To ensure that the frequent transposition of Tn10 was not due to the presence of *mutA* or *mutC*, we examined AZT‐resistant *tdk* knockouts in a strain from the same background, but without *mutA,C* and with a Tn10 inserted into *argE*. Here we sized the PCR products, using primers flanking the *tdk* gene, on a gel alongside of standards with and without insertions (Figure 3). Table 3 shows that indeed a majority of the *tdk* gene knockouts result from Tn10 insertions. To show that transpositions into *tdk* are not restricted to Tn10 or IS1, we also looked at *tdk* gene inactivations (AZT‐resistant mutants) in a strain (DE372) with a Tn5 insertion in the *umuC* gene. Table 3 presents the PCR product sizes that show that even with no selection for kanamycin resistance, half of the AZT‐resistant mutants have Tn5 insertions in *tdk*. (A full description of the strains used is given in Materials and Methods).

**TABLE 2:** Spontaneous mutations of *E. coli* using BW25113^a^ and CC107.^a^

Strain	BP	IS	In/Dels	Totals
CC107	26	7	8	41
BW25113	12	13	17	42

^a^ Data from Young et al. (2024).

**TABLE 3:** Transposons found within the *tdk* gene in *E. coli*.

Strain	Mutation	Occurrences
CC107 (*argE*::Tn10)	IS	5
	Tn5	0
	Tn10	16
	Base substitutions or indels	5
	Total	26
DE372 (*umuC*::Tn5)	IS	5
	Tn5	6
	Tn10	0
	Base substitutions or indels	1
	Total	12

### 3.3 Effect of Nucleoid Deficiency on Transposition

**FIGURE 4:**
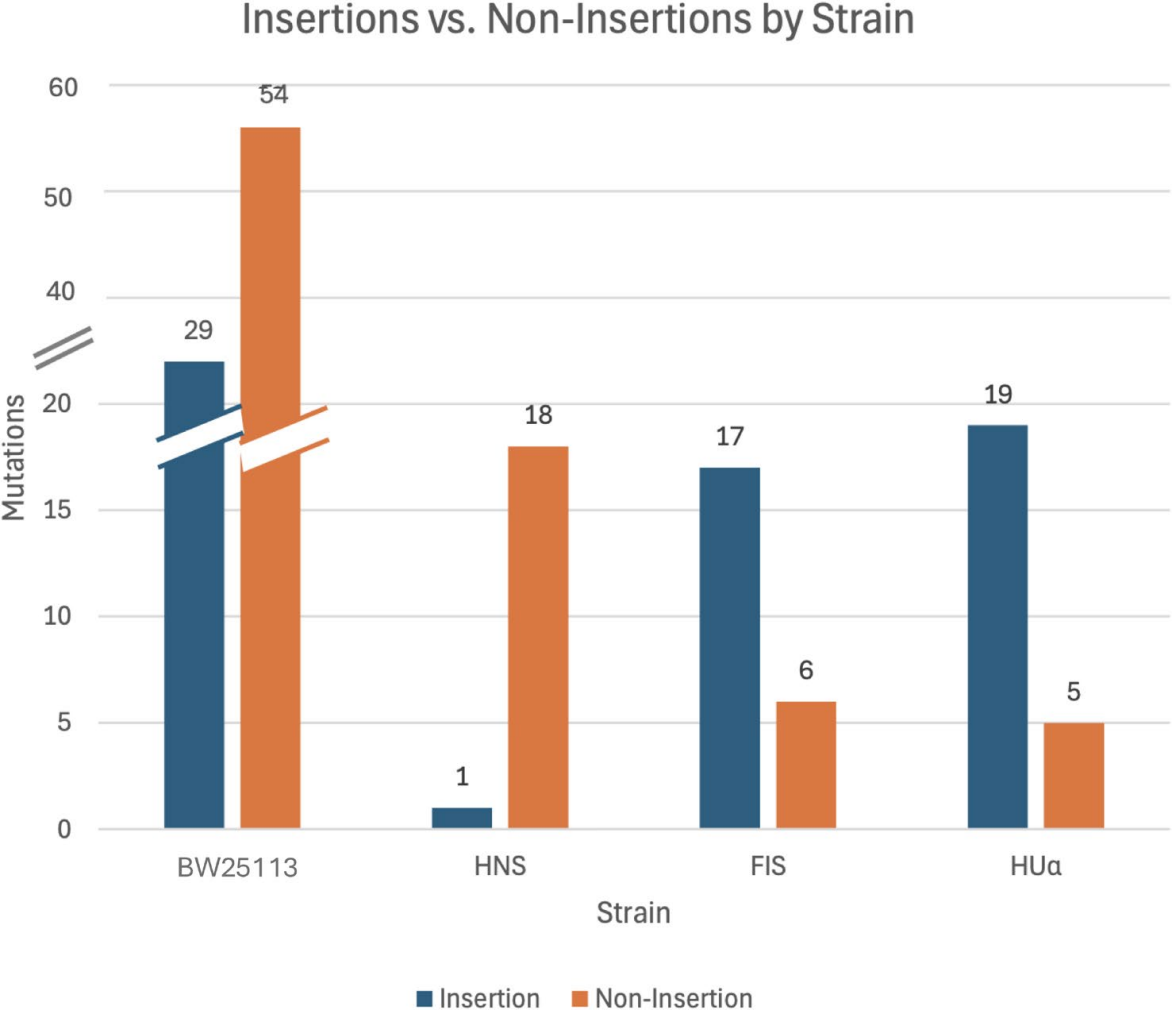
Insertions versus noninsertions in the *tdk* gene among spontaneous AZT‐resistant mutants in different derivatives of BW25113. The presence of insertion elements was determined by the size of PCR fragments (see Text). Thus, for BW25113 there were 54 insertions and 29 noninsertions among 83 mutants examined.

Bacterial DNA is highly compacted by a series of nucleoid proteins, FIS, HU (HUalpha, HUbeta), HNS, and IHF. Mutants deficient in any of the proteins can be expected to have some localized conformational changes of the DNA. We found that mutants deficient in FIS, IHF, or HUalpha have increased transpositions of IS1 relative to other types of mutations, whereas cells lacking HNS have decreased transposition (Figures 3 and 4; see also Shiga et al. 2001). We consider these findings in the Discussion.

### 3.4 Indels in the *tdk* Gene

**FIGURE 5:**
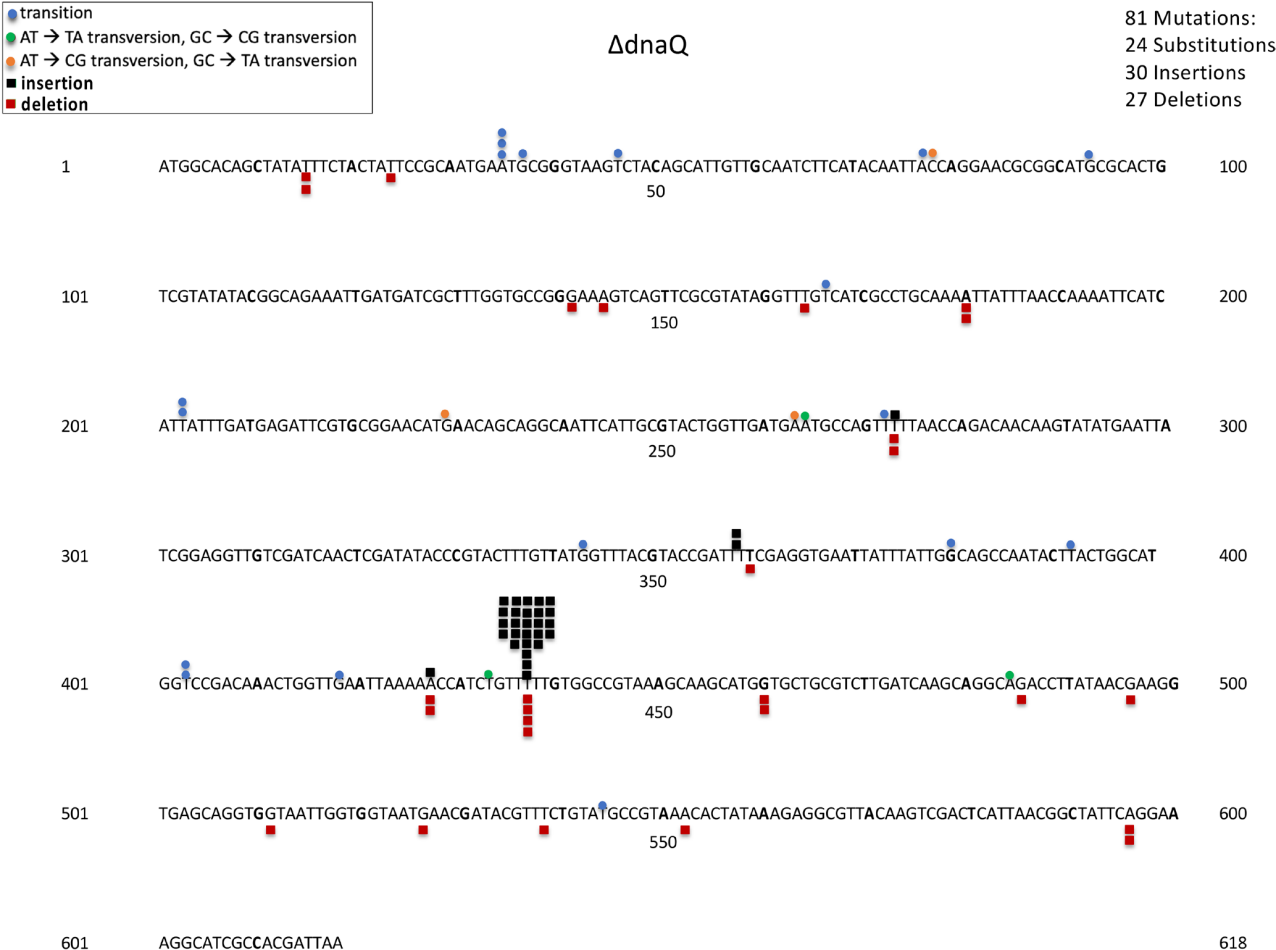
Distribution of spontaneous mutations detected in a *mutD* (*∆dnaQ*) derivative of BW25113. +1 insertions indicated by a black square, predominate between bp 435–439.

**FIGURE 6:**
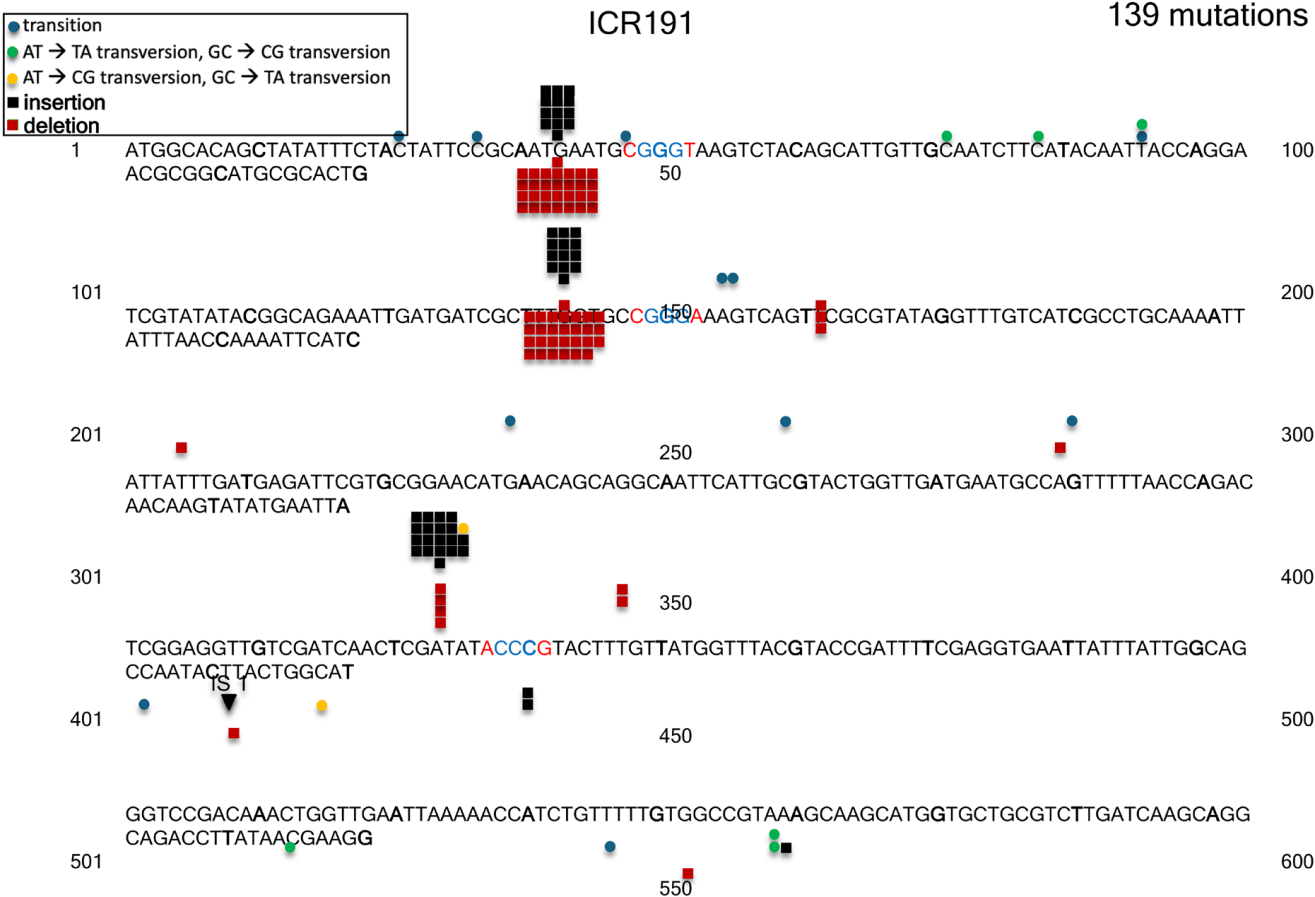
Distribution of 139 ICR191 induced mutations in *tdk*.

The *tdk* gene sequence has 3 runs of 5 monotonous A:T bp, and but 3 runs of 3 monotonous G:C bp. We looked at the mutator *mutD* (*∆dnaQ*) which lacks the epsilon subunit of DNA _polymerase, the editing function (Cox 1976; Echols et al. 1983). When grown in rich medium, the replication errors saturate the mismatch repair system (Schaaper and Radman 1989). Figure 5 depicts the sequence changes for 81 mutations in a *mutD* (*∆dnaQ)* strain. 57 of these are single bp insertions or deletions, and they show a hotspot at one of the three monotonous runs of A:T base pairs. In one case two ‐A‐ runs on opposite strands are very close together; beginning with base pair 423 the sequence on the nontranscribed strand is ‐A‐A‐A‐A‐A‐C‐C‐A‐T‐C‐T‐G‐T‐T‐T‐T‐T‐. There are only three occurrences at the first run, but 30 at the second, with additions outnumbering deletions 26:4. A third sequence, T‐T‐T‐T‐T‐ at bp 271 has only three occurrences. In contradistinction to *mutD*, ICR191 adds or deletes single bps at runs of ‐G‐'s (e.g., Calos and Miller 1980). There are only three runs of 3 ‐G‐'s in *tdk*, and no higher runs. Yet, ICR191 preferentially mutates these three runs, as seen in Figure 6. The different ratios of +1:−1 frameshifts at these sites remain an unexplained mystery, but are typical of ICR191 profiles (Calos and Miller 1980).

### 3.5 Further Analysis of Repair Deficient Strains

#### 3.5.1 alkB

**FIGURE 7:**
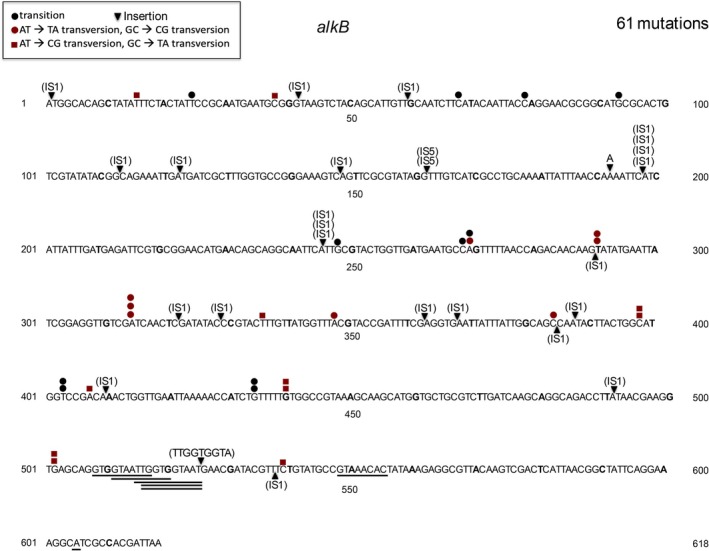
Distribution of 61 spontaneous mutations in an *alkB* derivative of BW25113. Underlined sequences represent deletions.

**FIGURE 8:**
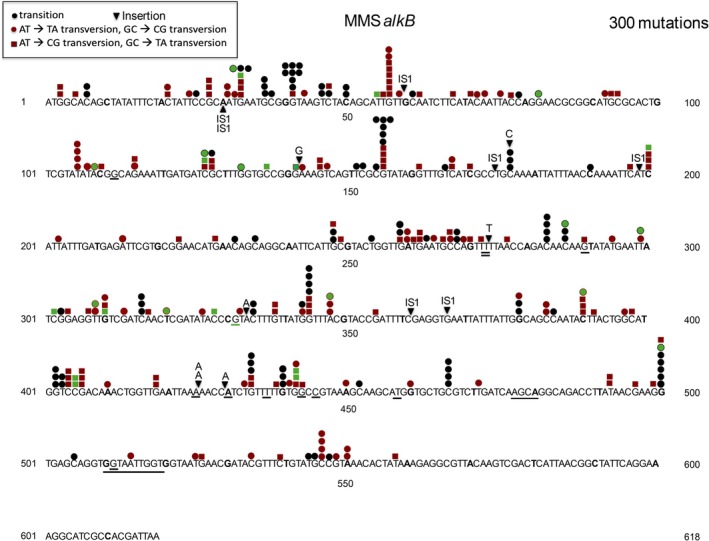
Distribution of 300 mutations induced by MMS in an *alkB* derivative of BW25113. Underlined sequences represent deletions.

We used the *tdk* reporter system to look at the spectrum of two different repair deficient strains. The *alkB* gene encodes a dioxygenase that oxidatively repairs 1‐methyladenine and 3‐methylcytosine lesions, both of which are generated by MMS (methyl methanesulfonate) (Trewick et al. 2002; Falnes et al. 2002; Begley and Samson 2003; Koivisto et al. 2003; Delaney and Essigmann 2004). Under normal conditions, *alkB* strains do not show up as mutators, as can be seen in Figure 7, which shows the distribution of 61 spontaneous mutations in *tdk*. Only 30 of 61 mutations are base substitutions, while 25 are IS insertions (23 IS1, 2 IS5). However, *alkB* strains are susceptible to mutagenesis by MMS (methyl methanesulfonate), as seen in Table 4, and much more so than wild‐type strains (Wrzesinski et al. 2010, and references therein). Although the base substitutions in an *alkB* strain treated with MMS have been reported (Wrzesinski et al. 2010), the reversion systems used are limited to either one or very few sites for each of the six base substitutions. Here, we show the full spectrum of mutations in the *tdk* gene that is able to monitor 345 different base substitution mutations at 248 different base pairs. Figure 8 shows the distribution of the mutations and Table 5 charts the number of occurrences of each type of base substitution and also the number of different sites represented. It is clear that the base changes are distributed among many sites, although G:C‐ > A:T and G:C‐ > T:A are the most prominent, with A:T ‐> T:A also somewhat well represented. The 243 base substitutions are distributed across 156 sites. No strong hotspots are evident.

**TABLE 4:** Mutant frequencies (*f*) of *E. coli* using an AlkB deficient strain and MMS treatment.

Strain	Condition	Time treated	Number of cultures	Frequency *f*(10^−8^)^a^ in *tdk*
AlkB	—	—	28	63 (49–93)
AlkB	30 μg/mL MMS	1.5 min	5	2650 (1770–3700)
AlkB	30 μg/mL MMS	3 min	14	5800 (2140–13,080)

*Note: tdk* mutant frequencies (*f*).

^a^ Values in parentheses are 95% confidence limits.

**TABLE 5:** Sites and mutations of *E. coli* *alkB* with MMS treatment (base pair substitutions only).

Base substitution	Sites	Sites found	Occurrences
G:C ➔ A:T	59	39	85
G:C ➔ T:A	81	53	79
A:T ➔ C:G	98	11	12
A:T ➔ T:A	58	30	37
G:C ➔ C:G	54	17	22
A:T ➔ G:C	28	6	8
Total	378	156	243

#### 3.5.2 sodA,B

**FIGURE 9:**
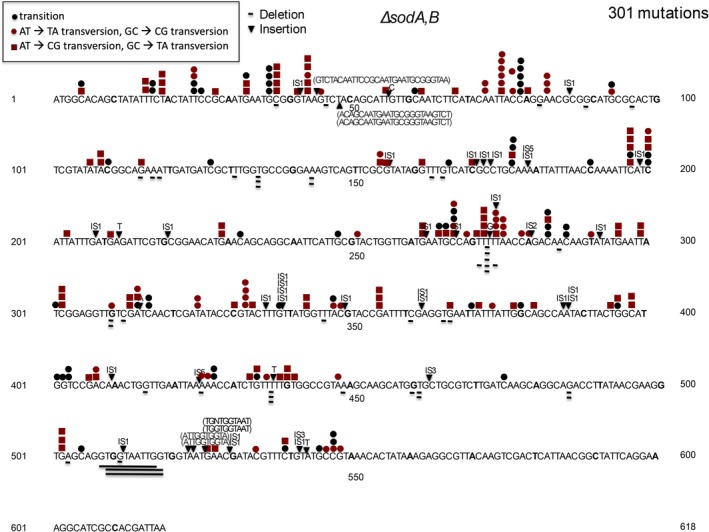
Distribution of 301 spontaneous mutations in *tdk* in a *sodA,B* derivative of MG1655. Underlined sequences represent deletions.

**FIGURE 10:**
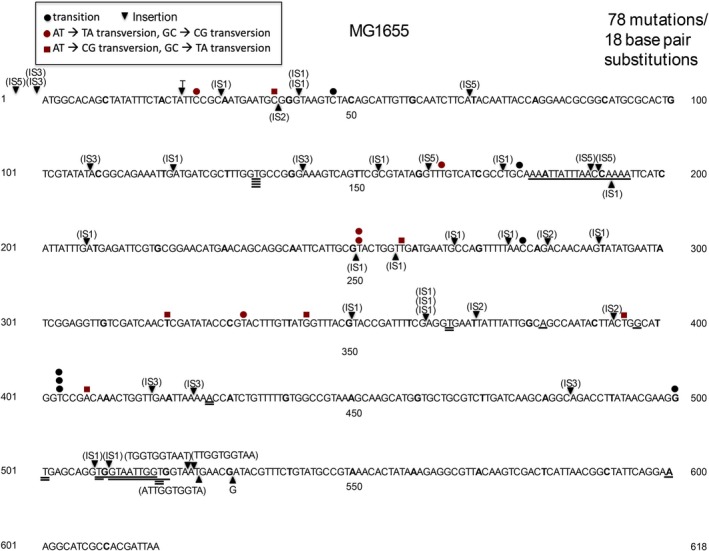
Distribution of 78 spontaneous mutations in *tdk* in the wild‐type strain MG1655. Underlined sequences represent deletions.

Double mutants lacking both superoxide mutases in *E. coli* are weak mutators, as revealed by genetic selection (Prieto‐Alamo et al. 1993), but the full spectrum of mutations caused in this case by unrepaired oxidative damage has not been reported. The *sodA,B* spectrum (Figure 9; Tables 6, 7) depicts 209 base substitutions, and 27 insertion elements (23 IS1, 1 IS2, 1 IS3, and 2 IS5). The insertion elements are seen in the spontaneous spectrum in *tdk* of both the specific wild‐type strain (MG1655; Figure 10), and also other wild‐type strains (e.g., Figure 4). Most of the base substitutions are unique to the *sodA,B* spectrum, and most or all of the IS insertions can be accounted for by the spontaneous background. However, there are also 31 single bp deletions and four single bp deletions, and six larger insertions that are partial duplications. It is not clear whether these are induced by the *sodA,B* defect or not.

## 4 Discussion

Seymour Benzer's classic studies in the rII region of phage T4 demonstrated that mutations did not occur randomly at all available sites, but instead occurred preferentially at certain sites termed “hotspots” (Benzer 1961). In some cases the hotspots were dramatically more mutation prone. Multiple studies form many different laboratories have shown that different factors may contribute to mutation rates for virtually all types of mutation, and particularly for hotspots. This is true for mutations occurring spontaneously, or resulting from the action of different mutagens. In some cases one factor may predominate, such as repeated sequence elements driving frameshift ‐like indels (Streisinger et al. 1966; Farabaugh et al. 1978; Pribnow et al. 1981), sequences signaling methylation of specific deoxycytidine residues (Coulondre et al. 1978), special secondary structures (Viswanathan et al. 2000), or context effects such as nearest neighbor bases (e.g., Coulondre and Miller 1977; Miller 1985; Horsfall et al. 1990; Rodriguez and Loechler 1991; Pienkowska et al. 1993; Nilsen et al. 1995; Bertenyi and Lambert 1996; Hatahet et al. 1998; Garibyan et al. 2003; Shee et al. 2012; Foster et al. 2018, see Discussion in Fernandez et al. 2020). Yet, some hotspots cannot be explained by these factors alone. In the study reported here, we have examined the hotspots among CPT‐induced mutations in the *tdk* gene of *E. coli* , and asked whether one extraordinary hotspot at bp 499 is a result of the DNA conformation resulting from the action of nucleoid binding proteins. Even in bacteria, the DNA is highly compacted by a series of nucleoid proteins that fold, and bend the chromosome (Kahramanoglou et al. 2011; Prieto et al. 2012; Johnson et al. 2021; Lioy et al. 2018). The most abundant of these proteins are FIS, HU (HUalpha, HUbeta), HNS, and IHF. Adhya and coworkers have extensively reviewed the recent finding of how these proteins, and others, effect looping, super‐coiling, compaction, and condensation of the *E. coli* chromosome (Verma et al. 2019). Cells lacking any of these proteins may have different conformations of the DNA in certain regions. For example, in HU‐deficient strains, the nucleoid is “decondensed,” as HU is involved in DNA compaction (Macvanin et al. 2012). IHF bends DNA (Rice et al. 1996), as does FIS (see Verma et al. 2019), HNS helps to form rigid filaments, and can act as a gene silencer (Winardhi et al. 2015). Here we show that the hotspot at position 499 virtually disappears (Figure 1) in strains lacking the HNS. Using genomic sequencing, Foster and coworkers (Foster et al. 2013, Niccum et al. 2019), have reported twofold increases in spontaneous mutations involving mainly base substitutions and indels in mutants lacking FIS or HU‐alpha in a mismatch repair deficient strain over the range of one half of the *E. coli* chromosome, but not the other half. However, they showed that loss of HNS does not yield such effects. That study and the current study complement each other in that one examined the length of the chromosome as a whole, and the other examined short regions in the 618 bp *tdk* gene. Also, our current study is focused on CPT‐induced mutations that result from CPT adducts on certain adjacent or near adjacent purines. Future studies are aimed at looking at the effect of a larger array of nucleoid protein mutants on specific hotspots in *tdk* by CPT and other mutagens.

Studies of transposable elements have shown that A:T rich regions are favored targets for transposition events (e.g., Tu and Cohen 1980). The A:T rich (58% A:T) *tdk* gene and its preceding 700 bp region (58% A:T) offer a favored target for transpositions of both IS elements and transposons (Figure 2, Tables 2 and 3). This provides a powerful assay for the movement of IS elements that are not marked with an antibiotic resistance gene. Thus, simple selection for AZT‐resistant TDK negative mutants reveals relatively high percentages of transpositions of either transposons or resident IS elements. This could be a tool for detecting transposable elements that carry no selectable markers from DNA libraries. We have used this assay to examine the effect of DNA conformation on transpositions by looking at mutants that lack one of the nucleoid binding proteins. As Figures 3 and 4 show, cells lacking FIS or HUalpha have increased transpositions of the resident IS elements relative to other types of mutations, and cells lacking HNS have decreased transpositions. The HNS results is in line with the work of Ohtsubo and coworkers (Shiga et al. 2001), who showed that IS1 transpositions are depended on a functional HNS protein (Tables 6 and 7).

**TABLE 6:** Mutant frequencies (*f*) of *E. coli* ∆sodA, B.

Strain	Number of cultures	Frequency *f* (10^−8^)^a^ in *tdk*
MG1655	10	614 (73–1136)
∆Sod A, B	11	1297 (533–4090)

*Note: tdk* mutant frequencies (*f*).

^a^ Values in parentheses are 95% confidence limits.

**TABLE 7:** Sites and mutations of *E. coli* using *sodA,B* (base pair substitutions only).

Base substitution	Sites	Sites found	Occurrences
G:C ➔ A:T	30	39	47
G:C ➔ T:A	33	53	58
A:T ➔ C:G	24	11	37
A:T ➔ T:A	19	30	38
G:C ➔ C:G	12	17	13
A:T ➔ G:C	9	6	16
Total	127	156	209

Genes that are A:T rich, such as *tdk*, may yield somewhat different spectra than those that are G:C rich. Figure 5 is a case in point. Here in a *mutD* strain that lacks the proof‐reading epsilon subunit of DNA polymerase III, mutations are predominantly found at a monotonous run of 5 A:T bp. In this case the plus 1 additions outnumber the −1 deletions 28:2. In a previous extensive study of *mutD*‐induced mutations in the first 200 bp of *lacI* in *E. coli* , base substitutions and −1 deletions at monotonous runs of A:T bp were most prevalent, although the system used could not detect +1 additions. In any case, the preference for +1 or −1 mutations at any particular site can vary from site to site (e.g., ICR191 induced frameshifts; Calos and Miller 1980). Interestingly, there are two other runs of 5 A:T bp in the gene that have very few occurrences in this sample. One run is just 5 bp away from the hotspot, so the nontranscribed strand shows the sequence ‐‐‐TTTTT‐‐‐‐AAAAA‐‐‐. The other run is at bp 248 and does have the 5 A's on the same strand as the hotspot, but has only two occurrences. Thus, even though one factor predominates, other factors still can play a determining role regarding mutation rates. Also, the ICR191 induced mutations in *tdk* target only monotonous runs of G:C bps (Figure 6), as predicted from earlier work (e.g., Calos and Miller 1980), even though the gene is A:T rich.

## 5 Conclusion

Nucleoid‐binding proteins that fold, compact, bend, and supercoil chromosomal DNA can impact mutation rates and, in the case reported here, can be the principal factor in determining hotspots.

## Author Contributions

Jeffrey H. Miller designed the study. Dana Sorensen, Tara Mata, Ananya Sridharan, Mallika Mathew, Emily Sagastume, Natasha Sutkin, Katherine Douglas, Mila Daniel, Angela Hung, Alina Garmash, Annette Hsieh, and Jamie Elizabeth Ortega performed the experiments and generated the figures. Jeffrey H. Miller wrote the manuscript. All authors reviewed the submitted version of the manuscript.

## Funding

This work was partially funded by a Faculty Research Grant from the University of California.

## Conflicts of Interest

The authors declare no conflicts of interest.

## Data Availability

Research data are not shared.
